# Identification of candidate anti-cancer molecular mechanisms of Compound Kushen Injection using functional genomics

**DOI:** 10.18632/oncotarget.11788

**Published:** 2016-09-01

**Authors:** Zhipeng Qu, Jian Cui, Yuka Harata-Lee, Thazin Nwe Aung, Qianjin Feng, Joy M. Raison, Robert Daniel Kortschak, David L. Adelson

**Affiliations:** ^1^ Department of Genetics and Evolution, School of Biological Sciences, The University of Adelaide, Adelaide, South Australia 5005, Australia; ^2^ Shanxi Modern Chinese Medicine Engineering Laboratory, Shanxi University of Traditional Chinese Medicine, Shanxi 030619, China

**Keywords:** systems biology, traditional Chinese medicine, lncRNA, transcriptome

## Abstract

Compound Kushen Injection (CKI) has been clinically used in China for over 15 years to treat various types of solid tumours. However, because such Traditional Chinese Medicine (TCM) preparations are complex mixtures of plant secondary metabolites, it is essential to explore their underlying molecular mechanisms in a systematic fashion. We have used the MCF-7 human breast cancer cell line as an initial *in vitro* model to identify CKI induced changes in gene expression. Cells were treated with CKI for 24 and 48 hours at two concentrations (1 and 2 mg/mL total alkaloids), and the effect of CKI on cell proliferation and apoptosis were measured using XTT and Annexin V/Propidium Iodide staining assays respectively. Transcriptome data of cells treated with CKI or 5-Fluorouracil (5-FU) for 24 and 48 hours were subsequently acquired using high-throughput Illumina RNA-seq technology. In this report we show that CKI inhibited MCF-7 cell proliferation and induced apoptosis in a dose-dependent fashion. We integrated and applied a series of transcriptome analysis methods, including gene differential expression analysis, pathway over-representation analysis, *de novo* identification of long non-coding RNAs (lncRNA) as well as co-expression network reconstruction, to identify candidate anti-cancer molecular mechanisms of CKI. Multiple pathways were perturbed and the cell cycle was identified as the potential primary target pathway of CKI in MCF-7 cells. CKI may also induce apoptosis in MCF-7 cells via a p53 independent mechanism. In addition, we identified novel lncRNAs and showed that many of them might be expressed as a response to CKI treatment.

## INTRODUCTION

The complexity of carcinogenesis at the genetic level has been investigated more and more deeply by leveraging fast-developing omics-related techniques in the past decades [1–3]. Novel genetic mutations and molecular markers are now comprehensively identified in cancer genome sequencing projects. More importantly, whole transcriptome analyses are much more widely used to identify novel cancer-related transcripts or regulatory elements, such as long non-protein-coding RNAs (lncRNAs) and alternative splicing, and are also used to characterise the underlying molecular mechanisms based on global gene expression changes in different types of cancers *in vivo* or *in vitro* [4–6]. The current challenge is to integrate these new techniques to discover or evaluate novel cancer therapies [7].

Traditional Chinese Medicines (TCMs) are experience-based remedies derived from hundreds or thousands of years of clinical use in China. Most TCMs are extracted from one or more medicinal herbs. The existence of multiple bioactive ingredients makes many TCMs potential novel resources for the discovery of new cancer drugs, such as multi-targeted cancer drugs [8]. Compound Kushen Injection (CKI, also known as Yanshu injection) is a State Administration of Chinese Medicine-approved TCM formula used in the clinical treatment of various types of cancers in China [9, 10]. It is extracted from the roots of two medicinal herbs, Kushen (*Radix Sophorae Flavescentis*) and Baituling (*Rhizoma smilacis Glabrae*), using modern standardised Good Manufacturing Processes (GMP) [11, 12]. The chemical fingerprint of CKI contains at least 8 different components, with primary compounds Matrine and Oxymatrine [12]. This indicates that multiple compounds in CKI may deliver an integrated anti-tumor effect through multiple targets and their associated molecular pathways.

By detecting the expression of key genes or proteins in single molecular pathways, the anti-tumor effects of Matrine or Oxymatrine, including the inhibition of cell proliferation and induction of apoptosis, have been demonstrated in various types of cancer [13–17]. The molecular mechanisms of CKI as a system have also been recently explored [11, 18]. Quantitative detection of expression changes of key regulators, including *beta-catenin*, *CyclinD1* and *c-Myc*, of the canonical Wnt/beta-catenin pathway, have shown that CKI can suppress the stem cells in MCF-7 cells by down-regulating this signalling pathway [11]. In addition, other studies suggest that CKI can inhibit mouse sarcoma growth and reduce tumor-induced hyperalgesia via the AKT and TRPV1 signalling pathways by reducing the phosphorylation of ERK and AKT kinases and BAD [18].

The main goal of modern pharmacology is to elucidate the molecular mechanisms that can be targeted by therapeutic compounds. Analyses using purified single components of TCM can be somewhat useful, but are limited when it comes to identifying integrated systemic effects resulting from a multi-compound formula. Furthermore, previous studies attempting to understand the mode of action of CKI have only focused on single or a few molecular pathways by assessing the expression of key regulators in these pathways. We have therefore, taken advantage of high throughput whole transcriptome analyses, and applied these to explore the system wide molecular mechanisms targeted by TCM. We have identified a comprehensive list of expressed genes perturbed by CKI, and used gene expression data to characterise molecular pathways potentially targeted by CKI in MCF-7 human breast cancer cells. Our results show that CKI can alter the expression of many cancer relevant genes and lncRNAs, correlated with the inhibition of cell proliferation through cell cycle arrest and the induction of apoptosis via p53 independent pathways.

## RESULTS

### CKI inhibits MCF-7 cell proliferation and induces cell apoptosis

**Figure 1:**
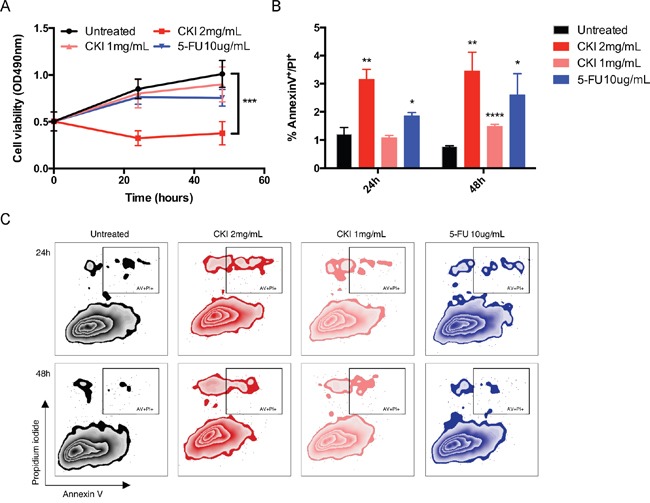
CKI inhibits proliferation and induces apoptosis of MCF-7 cells **A.** Inhibition of MCF-7 cell proliferation with CKI treatment. The level of viability of cells under different treatments was measured using XTT:PMS. Data are represented as mean ±SEM (n=6). **B.** and **C.** Induction of apoptosis in MCF-7 cells with CKI treatment. The level of apoptosis was determined by measuring the levels of Annexin V and PI staining: B) Percentages of Annexin V^+^/PI^+^ cells, and C) representative plots of Annexin V and PI staining. Data are represented as mean ±SEM (n=6). Statistical analyses were performed using A) two-way ANOVA or B) t-test comparing with “untreated” (*p<0.05, **p<0.01, ****p<0.0001).

To characterise the effect of CKI on proliferation of MCF-7 breast cancer cells, we used the XTT assay to measure cell viability after treating with different doses of CKI. The proliferation of MCF-7 cells was dramatically inhibited when treated with a high dose of CKI (2 mg/mL, based on the total alkaloid concentration in CKI) and showed a dose-dependent effect (Figure 1A and Supplementary Figure 1A). An Annexin V/Propidium Iodide (PI) assay was used to quantify cell apoptosis when MCF-7 cells were treated with CKI. Percentage of apoptotic cells, particularly at the higher dose of CKI, was increased at both time points compared with untreated cells, indicating that apoptosis was induced in cells treated with CKI (Figure 1B and 1C). The caspase3/7 colorimetric assay also showed that there was increased caspase3/7 activity in cells treated with CKI (Supplementary Figure 1B). Altogether, these results showed that CKI could inhibit growth and induce apoptosis of MCF-7 cells *in vitro*.

### Global gene expression changes in MCF-7 cells treated with CKI

**Figure 2:**
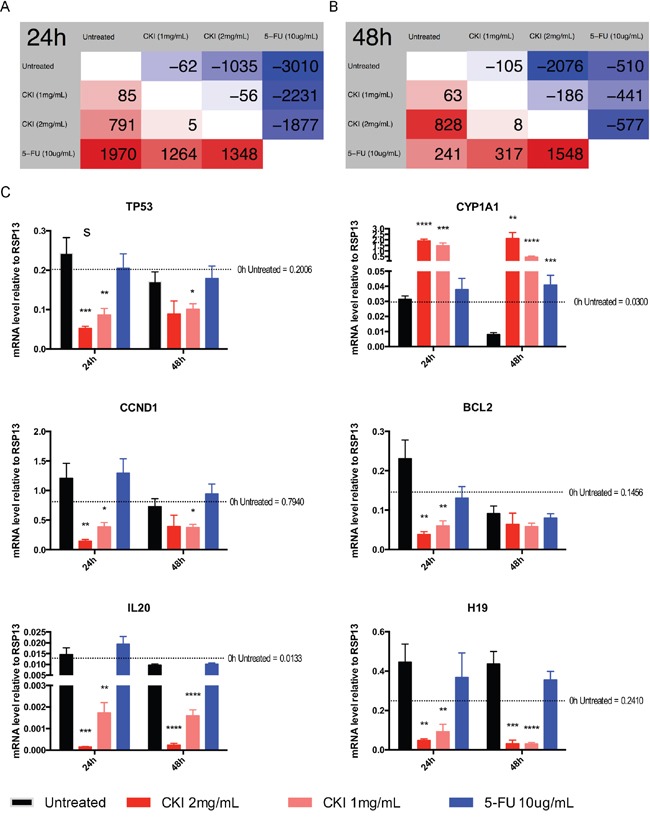
Differential gene expression in MCF-7 cells treated with CKI or 5-FU for 24 and 48 hours Numbers of DE genes (FDR < 0.05 according to edgeR) between different groups at **A.** 24 hours or **B.** 48 hours time. Comparison is based on row against column. Therefore, cells with a red background show numbers of up-regulated genes and cells with a blue background show numbers of down-regulated genes. **C.** Validation of transcriptome sequencing. Total of 6 DE genes (*TP53*, *CCND1*, *CYP1A1*, *BCL2*, *IL-20* and *H19*) identified by transcriptome sequencing were selected and were subjected to validation analysis by qPCR. Data are represented as mean ±SEM (n=9). Statistical analyses were performed using t-test comparing with “untreated” (*p<0.05, **p<0.01, ***p<0.001, ****p<0.0001).

To further investigate the underlying molecular mechanisms of CKI on MCF-7 cells, we performed high-depth next generation sequencing using an Illumina HiSeq 2500. In total, more than 732 million stranded 100 basepairs (bp) paired-end reads were sequenced from 9 groups of MCF-7 cells treated with two doses of CKI or one dose of chemotherapy drug 5-Fluorouracil (5-FU) for 24 and 48 hours along with untreated cells (Supplementary Table 1) (GSE78512). The global gene expression profiles of CKI treated cells, particularly in cells treated with high dose CKI (2 mg/mL), were clearly different from the profile of 5-FU treated cells compared with untreated cells (Supplementary Figure 2). We then used edgeR to identify the statistically significant differentially expressed (DE) genes for the pairwise comparisons between cells treated with 1 mg/mL CKI, 2 mg/mL CKI and 5-FU for 24 or 48 hours respectively (Figure 2 and Supplementary Table 2). Compared with untreated cells, fewer than 200 genes had significantly altered expression in cells treated with low dose CKI (1 mg/mL) for 24 or 48 hours (Figure 2A and 2B). However, many more DE genes (1,826 genes for 24 hours and 2,904 for 48 hours) were identified in cells treated with high dose CKI (2 mg/mL). Interestingly, when comparing the number of DE genes in cells treated with high dose CKI with low dose CKI, we observed almost twice as many genes down-regulated but only a small number of genes (828 to 791) up-regulated (Figure 2A and 2B). Furthermore, we compared DE genes in cells treated with high dose CKI and in cells treated with 5-FU (Supplementary Figure 3). For up-regulated genes in cells treated with high dose CKI for 24 hours, approximately half (396 out of 791) of these were also identified as DE genes, with most (384) being up-regulated in 5-FU treated cells as well. 459 down-regulated genes were also shown as DE genes with most of these (424 out of 459) also being down-regulated in cells treated with 5- FU for 24 hours (Supplementary Figure 3A). After cells were treated with CKI or 5-FU for 48 hours, the number of DE genes decreased dramatically in 5-FU treated cells, but greatly increased in cells treated with 2 mg/mL CKI (Supplementary Figure 3B). The common DE genes altered by CKI or 5-FU showed consistent expression changes at 48 hours (Supplementary Figure 3B).

To validate the gene expression changes from transcriptome analysis, we performed quantitative PCR (qPCR) for 6 genes and acquired overall consistent results (Figure 2C, Supplementary Figure 4 and Supplementary Table 2).

### Annotation of the molecular pathways altered by CKI in MCF-7 cells

Since CKI likely contains multiple bioactive ingredients, we used a number of systems biology methods to explore the molecular mechanisms of CKI.

**Figure 3:**
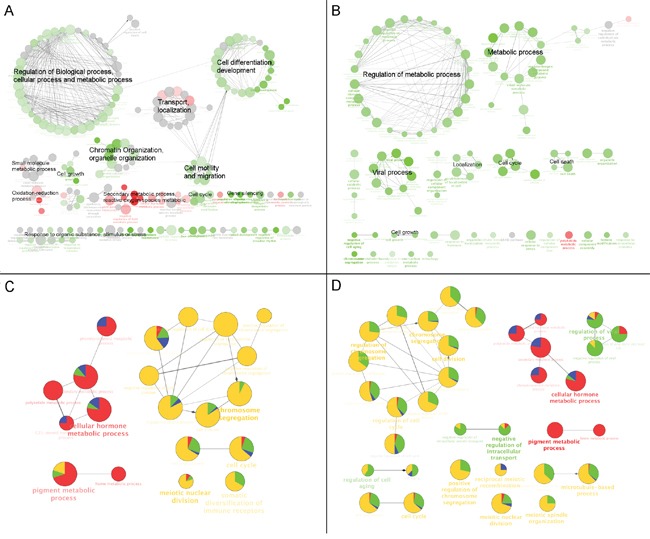
GO functional annotation of DE genes in CKI treated cells *Over-represented GO terms (Biological Process at 3rd level) for DE genes identified from comparison of CKI treated cells against untreated cells for* **A.** *24 hours or* **B.** *48 hours*. Red coloured nodes mean more than 60% of DE genes that contributed to a term were up-regulated and green coloured nodes mean more than 60% of DE genes that contributed to a term were down-regulated. The colour gradient represents the proportion of up- or down- regulated genes between these cut offs, and the node size is proportional to the significance of over-representation. Terms with similar functional classifications are connected with edges and the most significant term in each cluster is shown in bold. *Comparison of over-represented GO terms for the top 200 significant DE genes in cells treated with 2 mg/mL CKI or 5-FU for* **C.** *24 hours or* **D.** *48 hours.* Four different colours were used to represent the proportion of DE genes from up- or down- regulated genes. For CKI (red = up-regulated and green = down-regulated) or 5-FU (blue = up-regulated and yellow = down-regulated). Node size is proportional to the significance of over-representation and terms with similar functional classifications are connected with edges and the most significant term in each cluster is shown in bold.

The over-represented Gene Ontology (GO) terms for all DE genes identified in cells treated with high dose CKI (2 mg/mL) for 24 hour and 48 hours are shown in Figure 3A and 3B. Based on their functional similarity, these GO terms were clustered into several primary categories, including “Regulation of biological process, cellular process and metabolic process”, “Cell differentiation, development”, “Transport, localisation”, “Chromatin organisation, organelle organisation”, “Cell motility and migration” and “Secondary metabolic processes and reactive oxygen species metabolic”. Interestingly, we found that the majority of cell growth or proliferation related GO terms included more down-regulated genes, while GO terms associated with “Secondary metabolic processes and reactive oxygen species metabolic” showed enrichment of more up-regulated genes (Figure 3A). In MCF-7 cells treated with CKI for 48 hours, similar categories of over-represented GO terms seen at 24 hours were also observed, such as “Metabolic process”, “Regulation of metabolic process” and “Localization”. In addition, cell proliferation related terms, including “Cell cycle”, “Cell growth” and “Cell death” were also over-represented in DE genes from cells treated with CKI for 48 hours (Figure 3B). Furthermore, we compared the over-representation of GO terms of the 200 most significantly DE genes in cells treated with CKI or 5-FU for 24 hours or 48 hours (Figure 3C and 3D). In cells treated with CKI or 5-FU for 24 hours, over-represented GO terms were generally divided into two clusters with respect to the different expression status of the genes that contributed to each term. Terms such as “Cellular hormone metabolic process” and “Pigment metabolic process” were dominated by up-regulated genes, which were mainly DE genes from CKI treated cells only. On the other hand, terms represented by “Chromosome segregation”, “Cell cycle”, “Meiotic nuclear division” and “Somatic diversification of immune receptors”, were mainly contributed by down-regulated genes, particularly DE genes from 5-FU treated cells. After 48 hours, the same over-represented GO terms in cells treated with CKI or 5-FU for 24 hours were found, but the proportions of DE genes from cells treated with CKI were increased for most of these terms, particularly for “Chromosome segregation” related terms. In addition, more significantly over-represented GO terms were found in cells treated with CKI or 5-FU for 48 hours compared to those in 24 hours, such as “Regulation of viral process” and “Negative regulation of intracellular transport”, and the majority of DE genes that contributed to these terms were down-regulated in cells treated with CKI.

**Figure 4:**
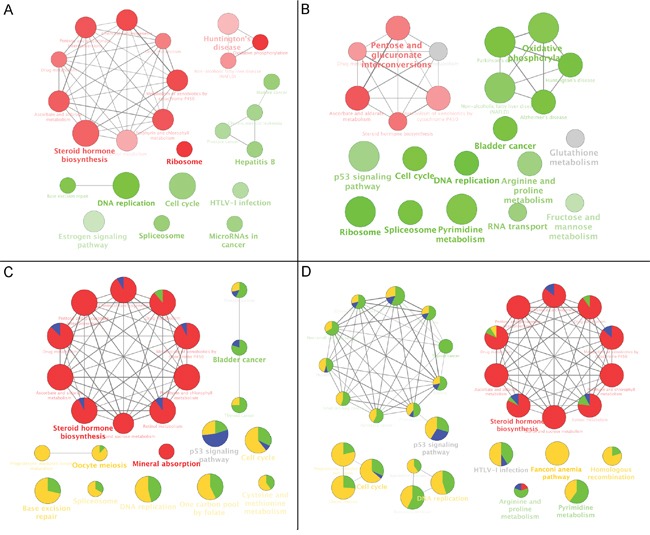
KEGG functional annotation of DE genes in cells treated with CKI *Over-represented KEGG pathways for all DE genes identified from comparison of CKI treated cells with untreated cells for* **A.** *24 hours or* **B.** *48 hours*. Red coloured nodes mean that more than 60% of DE genes that contributed to this pathway were up-regulated and green coloured nodes mean that more than 60% of DE genes that contributed to this pathway were down-regulated. The colour gradient represents the proportion of up- or down- regulated genes between these two cut offs, and node size is proportional to the significance of over-representation. Pathways with similar functional classifications are connected with edges and the most significant term in each cluster is shown in bold. *Comparison of over-represented KEGG pathways for the top 200 significant DE genes in cells treated with 2 mg/mL CKI or 5-FU for* **C.** *24 hours or* **D.** *48 hours*. Four different colours were used to represent the proportion of DE genes from up- or down- regulated genes. For CKI (red = up-regulated and green = down-regulated) or 5-FU (blue = up-regulated and yellow = down-regulated). Node size represents the significance of over-representation and terms with similar functional classifications are connected with edges and the most significant term in each cluster is shown in bold.

In order to further characterise the potential functional pathways altered by CKI, we performed over-representation analysis of Kyoto Encyclopedia of Genes and Genomes (KEGG) pathways for all DE genes in cells treated with high dose CKI. Metabolic pathways represented by “Steroid hormone biosynthesis”, and including “Pentose and glucuronate interconversions” and “Drug metabolism” and so on, were over-represented based on DE genes in cells treated with CKI for 24 hours (Figure 4A). The majority of DE genes that contributed to these terms were up-regulated (Figure 4A). Over-represented cell growth related pathways, such as “Cell cycle” and “DNA replication”, were also observed (Figure 4A). In addition, cancer-related pathways, such as “Prostate cancer”, “Bladder cancer” and “MicroRNA in cancer”, were also shown as over-represented pathways. It is also interesting to note that DE genes that contributed to cell growth and cancer related pathways were generally down-regulated in cells treated with CKI (Figure 4A). After cells were treated with CKI for 48 hours, most of the over-represented pathways found at 24 hours were still shown as significantly over-represented. However, some over-represented metabolic pathways and disease-related pathways at 48 hours were not shown as significantly over-represented pathways in cells treated with CKI for 24 hours. These pathways included “Arginine and proline metabolism”, “Pyrimidine metabolism”, “Fructose and mannose metabolism”, “Parkinson's disease” and “Alzheimer's disease”. In contrast to over-represented metabolic or disease related pathways in cells treated with CKI for 24 hours, these 48-hours-only significant over-represented metabolic or disease pathways were mostly a function of down-regulated DE genes (Figure 4B). Next, we compared the over-represented KEGG pathways based on the top 200 significantly DE genes in cells treated with CKI or 5-FU. Consistent with the results in Figure 4A and 4B, metabolic related pathways were primarily contributed by CKI up-regulated genes. Cell growth and cancer related pathways were also over-represented, and were mostly contributed by down-regulated genes in cells treated with CKI or 5-FU (Figure 4C and 4D). More significantly over-represented cancer-related pathways were found in cells treated with CKI or 5-FU after 48 hours, and DE genes in these pathways were mainly down-regulated (Figure 4D).

### Many pathways perturbed by CKI in MCF-7 cells were inhibited

**Figure 5:**
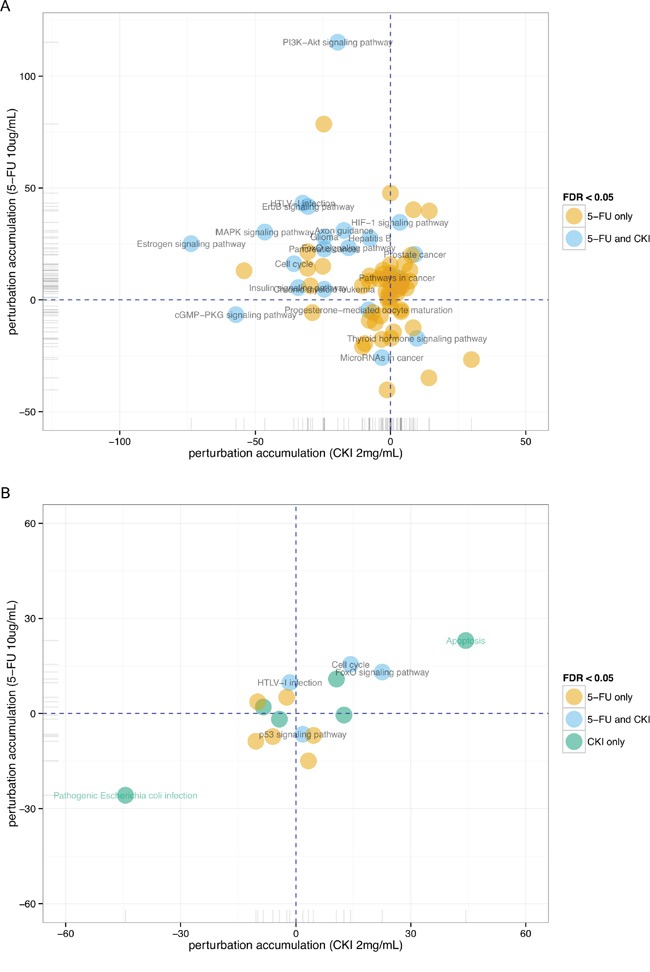
Perturbation of KEGG pathways in cells treated with CKI or 5-FU for **A.** 24 hours or **B.** 48 hours inferred with SPIA. Perturbation accumulation and significance of perturbation for each KEGG pathway were calculated based on the fold changes of expression of DE genes compared to untreated cells, integrated with the topology information in this pathway. Positive perturbation accumulation values mean this pathway is activated and *vice versa*. “5-FU only” or “CKI only” represent pathways that are only significantly perturbed in one condition not in the other.

From the above gene set enrichment analysis, we observed that many over-represented GO terms or KEGG pathways were enriched in down-regulated genes from cells treated with CKI. We used Signalling Pathway Impact Analysis (SPIA) to identify significantly perturbed functional pathways when integrating gene expression information with signalling pathway topology [19]. 21 KEGG pathways were identified as significantly perturbed in cells treated with high dose CKI (2 mg/mL) after 24 hours, and the majority of these pathways (16 out of 21) were shown as inhibited (Supplementary Table 3). In cells treated with 5-FU for 24 hours, more KEGG pathways (75) were identified as significantly perturbed, but only 22 of these were shown as inhibited (Supplementary Table 3). We then compared these significantly perturbed pathways in cells treated with CKI or 5-FU. Interestingly, all significantly altered pathways in cells treated with CKI were also shown as significantly perturbed in cells treated with 5-FU (Figure 5A). This suggests that at 24 hours, CKI and 5-FU perturbed some of the same pathways. However, the perturbation status of these common altered pathways in cells treated with CKI or 5-FU was quite different. The majority of inhibited pathways in cells treated with CKI were shown as activated in cells treated with 5-FU (Figure 5A). Although the perturbation status indicated by SPIA is just suggestive, it still provides some clues that CKI might target different genes even though it might perturb the same pathway as 5-FU. After cells were treated with CKI or 5-FU for 48 hours, 11 significantly perturbed pathways were identified in each of treatment group, but only 3 of these were shown as significantly perturbed pathways in both cells treated with CKI or 5-FU (Figure 5B).

**Figure 6:**
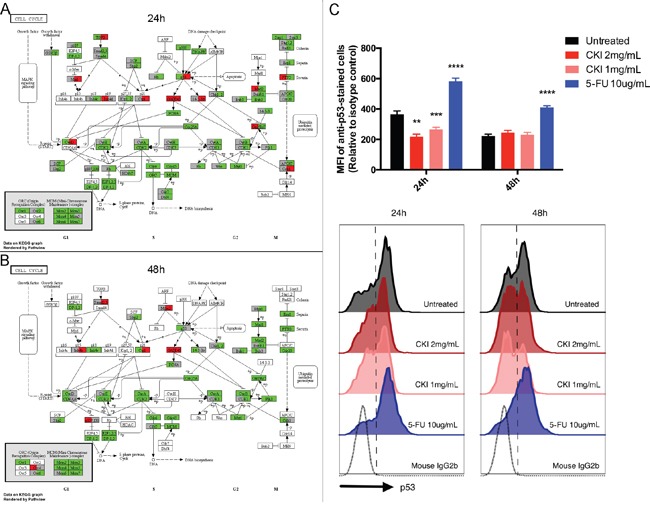
Comparison of individual gene expression change in MCF-7 cells treated with CKI (2 mg/mL) or 5-FU for **A.** 24 hours or **B.** 48 hours in the cell cycle pathway. Significant DE genes are coloured with red (up-regulated) or green (down-regulated). Each coloured box is separated into two parts, the left half represents the expression change status in cells treated with CKI and the right half represents the expression change status in cells treated with 5-FU. White or grey colours represent gene(s) that are not significantly differentially expressed. **C.** CKI caused down-regulation (24 hours) or no significant change (48 hours) of p53 protein level. The level of p53 protein present in cells treated with CKI was measured by flow cytometry. For the top panel, mean fluorescent intensity (MFI) of cells stained with anti-p53 where MFI of isotype control was subtracted. Data are represented as mean ±SEM (n=9). Statistical analyses were performed using t-test comparing with “untreated” (**p<0.01, ***p<0.001, ****p<0.0001). The bottom panel shows representative histograms of anti-p53 staining.

In order to examine the perturbation of CKI on KEGG pathways at the individual gene level, we mapped the expression status of DE genes in cells treated with CKI or 5-FU on the cell growth and death related pathway “Cell cycle” as an example (Figure 6). Consistent with what we observed in the above KEGG over-representation analyses, the majority of DE genes in the “Cell cycle” pathway were down-regulated both in cells treated with CKI or 5-FU. Many essential regulators, such as *Cyclin-dependent kinase 2* (*CDK2*), *Transcription Factor Dp-1* (*DP-1*), *Origin recognition complex* (*ORC*) and *Minichromosome maintenance protein complex* (*MCM*) families, which are important in regulation of the G1/S transition [20, 21], were significantly down-regulated both in cells treated with CKI or 5-FU. However, some key regulators in this “Cell cycle” pathway had different expression status in cells treated with CKI or 5-FU. For example, *CCND1*, which encodes Cyclin-D1 (a member of the CycD protein family), was significantly down-regulated in cells treated with CKI compared with untreated cells. In contrast, *CCND3*, encoding Cyclin-D3 which also belongs to the CycD protein family, was significantly up-regulated in cells treated with 5-FU. Interestingly, as an important pro-apoptosis modulator, the expression of *p53* was opposite in cells treated with CKI (down-regulated) or 5-FU (up-regulated) compared to untreated cells. In addition, the protein levels of p53 were significantly decreased in cells treated with CKI for 24 hours and showed no significant change at 48 hours. In contrast p53 increased in cells treated with 5-FU for both 24 and 48 hours (Figure 6C). Taken together the results of down-regulated p53 mRNA and protein levels (Figure 2C and 6C) but elevated apoptosis activity (Figure 1B) in MCF-7 cells treated with CKI, suggest that CKI may induce cell apoptosis in a p53 independent fashion. In cells treated with CKI or 5-FU for 48 hours, essential genes for G1/S transition (as discussed above) were still shown as significantly down-regulated. In addition, more genes, such as *Cyclin A1* (*CCNA1*, encoding CycA), *Cyclin B1 and B2* (*CCNB1* and *CCNB2*, encoding CycB), *Mitotic Arrest Deficient 1* (*MAD1*, encoding mad1) and *Mitotic Arrest Deficient 2* (*MAD2*, encoding mad2), which are important regulators of G2 or M phase, were shown as significantly down-regulated in cells treated with CKI for 48 hours.

The cell cycle assay using flow cytometry indicated that proportions of cells in G1 and S phases were significantly lower in MCF-7 cells treated with high dose CKI (2 mg/mL), while significantly higher in G2/M phase, indicating a cell cycle arrest at G2/M phase by CKI in MCF-7 cells (Supplementary Figure 5). In summary, possible p53 independent apoptosis together with perturbation of other cancer cell growth associated pathways, such as “Cell cycle”, probably contribute the anti-cancer effect of CKI.

### The expression of many clinically relevant cancer genes was altered in MCF-7 cells treated with CKI

**Figure 7:**
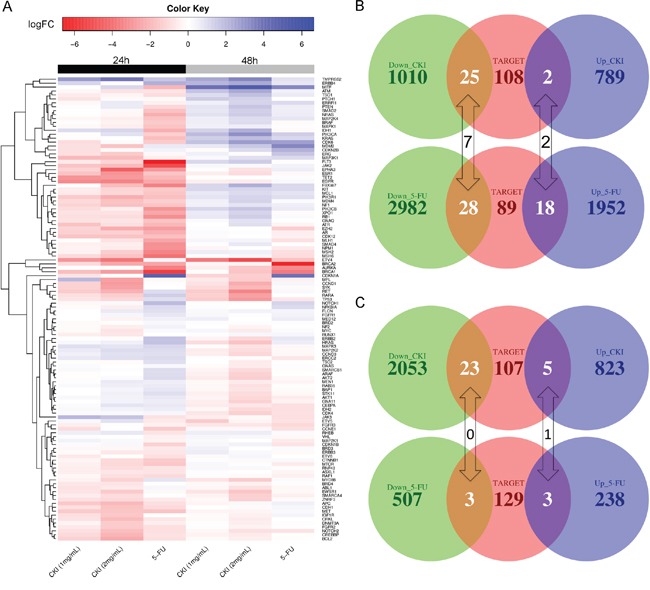
Expression change of clinically relevant cancer genes in cells treated with CKI or 5-FU **A.** Heatmap showing the expression fold changes of cancer relevant genes from the TARGET database. Overlap of TARGET genes with DE genes in MCF-7 cells treated with 2 mg/mL CKI or 5-FU for **B.** 24 hours or **C.** 48 hours.

To investigate the potential molecular targets of CKI in MCF-7 cells, we examined the changes in expression of 135 genes in a curated database of Tumour Alterations Relevant for Genomics-driven Therapy (TARGET) from The Broad Institute (https://www.broadinstitute.org/cancer/cga/target). These genes are directly linked to a clinical outcome when somatically altered in cancer. Many genes showed similar expression changes in cells treated with CKI or 5-FU, and this confirmed what we observed in the pathway analysis (see above) (Figure 7A). However, the expression of some genes was either of a higher degree or in a different direction in cells treated with CKI compared with cells treated with 5-FU. For example, *ETS translocation variant 4* (*ETV4*), whose overexpression is oncogenic in prostate cells [22], was greatly down-regulated in cells treated with CKI compared with cells treated with 5-FU. On the other hand, *Cyclin-Dependent Kinase Inhibitor 1A* (*CDKN1A*, also named as *p21*) was highly up-regulated in 5-FU treated cells but not in CKI treated cells. We then examined how many significantly DE genes in cells treated with CKI or 5-FU were also in this TARGET gene list. In total, 27 DE genes in cells treated with CKI for 24 hours were in the TARGET gene list, and more up-regulated genes (18 compared to 2) in cells treated with 5-FU for 24 hours were in the TARGET gene list (Figure 7B). In cells treated with CKI or 5-FU for 48 hours, 28 DE genes were in the TARGET list from cells treated with CKI, while only 6 of the DE genes from cells treated with 5-FU for 48 hours were in this list (Figure 7C).

### Reconstruction of non-coding and protein-coding RNA co-expression networks altered by CKI

**Figure 8:**
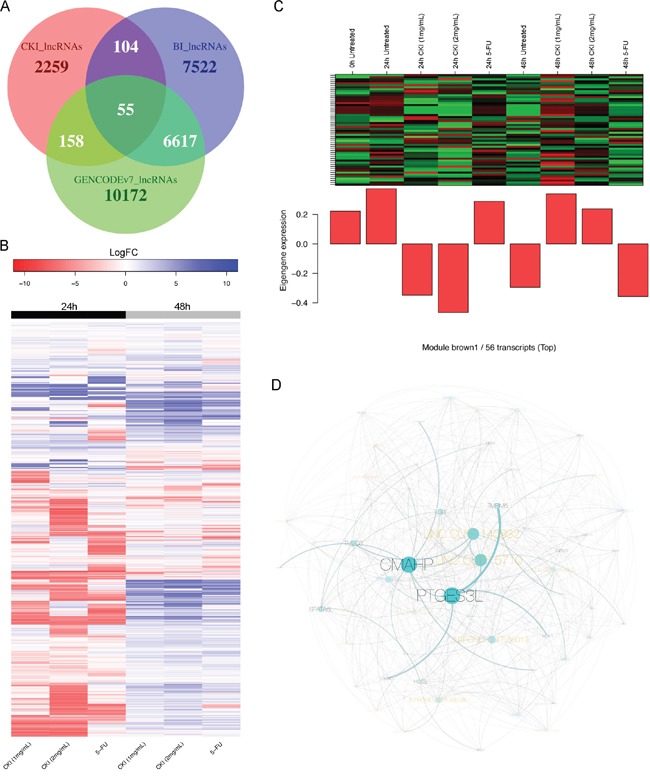
Expression change of *de novo* identified lncRNAs and an example of a CKI-specific co-expression module **A.** Overlap of *de novo* identified lncRNAs (CKI_lncRNAs) with two annotated human lncRNA datasets: “BI_lncRNAs” as annotated lncRNAs from the Broad Institute and “GENCODEv7_lncRNAs” as lncRNAs from GENCODE version7. **B.** Heatmap showing expression fold change of 2,287 lncRNAs in 6 treated cell samples compared to corresponding untreated cells. **C.** Expression pattern of transcripts in CKI-specific module “brown1” is shown in top panel, and barplot in bottom panel shows the eigengene values in different samples. Green represents “under-expressed” and red represents “over-expressed” in the heatmap. The “eigengene value” is defined as the first principal component of this module, so it can be considered as a representative of the gene expression profiles in this module. **D.** Visualization of CKI-specific co-expression module “brown1”. The black labels represent refGenes and gold labels represent lncRNAs. The size of the node/label and edge weight are proportional to between-ness betweeness centrality.

The differential expression analysis of refGenes showed that lncRNA, *H19*, was significantly down-regulated after cells were treated with CKI (Figure 2C and Supplementary Table 2). In order to better understand the potential expression change of lncRNAs in response to CKI in MCF-7 cancer cells, we carried out *de novo* identification of lncRNAs from this transcriptome dataset. In total, 2,576 lncRNA transcripts, which are from 2,287 unique genomic loci, were identified (Supplementary Figure 6). We also found that the majority of these lncRNAs were novel by comparing the genomic coordinates of these lncRNAs with two well-annotated human lncRNA datasets (Figure 8A) [23, 24]. The expression of many lncRNAs was changed in cells treated with CKI or 5-FU (Figure 8B). The expression of lncRNAs in cells treated with CKI for 24 hours was quite different compared with cells treated with 5-FU for 24 hours. While at 48 hours, we observed more similar lncRNA expression in cells treated with CKI or 5-FU (Figure 8B). These results indicate that some of these lncRNAs may play specific regulatory roles responding to different reagent treatments.

In order to identify potential lncRNA candidates that were highly relevant to CKI treatment in MCF-7 cells, we reconstructed the co-expression networks for 15,115 detectable refGenes and 2,287 lncRNAs in 9 different samples. 53 co-expression modules were reconstructed based on the expression profiles of refGenes or lncRNAs across 9 samples (Supplementary Figure 7 and Supplementary Table 4). Upon examination of the eigengene expression patterns of these 53 modules, we found three modules with expression profiles that were consistent with CKI-specific modules (Figure 8C and Supplementary Figure 8). Centrality analysis of these CKI-specific co-expression modules allowed us to identify “hub” nodes, many of which belonged to lncRNAs (Figure 8D and Supplementary Figure 8). These results showed that lncRNAs may be co-regulated or involved in the same regulatory pathways with protein-coding genes when MCF-7 cells are treated with CKI. We then performed GO and KEGG over-representation analysis for the protein-coding genes in these three CKI-specific modules, and “Cell proliferation” was identified as the most significantly over-represented functional term (Table 1). Interestingly, we also found genes in these CKI-specific co-expression modules are over-represented in functions involved in “intracellular signal cascade” and “second-messenger-mediated signaling” (Table 1), which might be triggered by the multiple molecular species present in CKI. In addition, we also identified three co-expression modules, which showed highly correlated co-expression profiles in both cells treated with CKI or 5-FU. We defined these as “CKI-5FU” modules (Supplementary Figure 9). These modules contained over-represented genes in “cell cycle”, “DNA replication” and other cell growth related pathways (Supplementary Table 5). This result was consistent with what we observed in our DE analysis (see above).

**Table 1:** Significantly over-represented GO and KEGG terms in protein-coding genes from three CKI-specific co-expression modules (count >4 and P-value < 0.05)

Category	Term	Count	Fold enrichment	P-value
**GOTERM_BP_FAT**	GO:0008283∼cell proliferation	8	3.354	0.009
**GOTERM_BP_FAT**	GO:0010604∼positive regulation of macromolecule metabolic process	11	2.346	0.016
**GOTERM_BP_FAT**	GO:0032989∼cellular component morphogenesis	7	3.223	0.020
**GOTERM_BP_FAT**	GO:0048514∼blood vessel morphogenesis	5	4.332	0.027
**GOTERM_BP_FAT**	GO:0035295∼tube development	5	4.155	0.031
**GOTERM_BP_FAT**	GO:0007242∼intracellular signaling cascade	13	1.892	0.036
**GOTERM_BP_FAT**	GO:0019932∼second-messenger-mediated signaling	5	3.890	0.038
**GOTERM_BP_FAT**	GO:0000904∼cell morphogenesis involved in differentiation	5	3.746	0.043
**GOTERM_BP_FAT**	GO:0000902∼cell morphogenesis	6	3.081	0.043
**GOTERM_BP_FAT**	GO:0001568∼blood vessel development	5	3.731	0.043
**GOTERM_BP_FAT**	GO:0001944∼vasculature development	5	3.642	0.047
**KEGG_PATHWAY**	hsa05200:Pathways in cancer	7	3.391	0.013
**KEGG_PATHWAY**	hsa04020:Calcium signaling pathway	5	4.514	0.021

## DISCUSSION

Many TCMs or other herbal medicines, such as CKI used in this study, are extracted from multiple medicinal herbs. The fact that multiple potentially bioactive ingredients are present in these formulas suggests that they have multiple targets, and therefore perturb multiple biological pathways. Transcriptome analysis using high-throughput next generation sequencing technologies has been widely used in cancer biology for the identification of novel biomarkers, mutations and even novel transcripts, such as lncRNAs, in different cancer types [4, 5, 25, 26]. We have used whole transcriptome analysis to identify potential molecular mechanisms of CKI *in vitro*. We not only identified potential gene targets of CKI based on differential gene expression, but also characterised potential biological pathways targeted by CKI. Although further experiments are required to validate these candidate targets, our results provide a very important starting point for subsequent experimental functional validation.

We have identified genes whose expression was significantly altered in MCF-7 cells treated with CKI. Consistent with the phenotypic analyses, the global gene expression changes in cells treated with CKI support a dose-dependent effect on MCF-7 cells, which had been reported in MCF-7 stem cells and other types of cancer cells in previous studies [10, 11]. However, we have generated a far more comprehensive candidate gene list by considering CKI as a whole rather than looking at the effect of individual constituents. As expected, genes, including *Cytochrome P450 family 1* (*CYP1A1*), *Aldo-Keto Reductase Family 1*, *Member C2* (*AKR1C2*) and *Member C3* (*AKR1C3*), which are involved in xenobiotic compound metabolism, were significantly up-regulated when cells were treated with CKI [27, 28] but not with 5-FU. On the other hand, many genes involved in cell growth or used as biomarkers of carcinogenesis, such as *CCND1* [29], were significantly down-regulated. Interestingly, we observed one lncRNA *H19*, known to be over-expressed in several types of cancer [30, 31], was dramatically down-regulated in cells treated with CKI. Recently, many lncRNAs have been characterised as important gene regulators in various types or stages of carcinogenesis [32]. We hypothesise that lncRNAs may be also involved in the gene regulatory networks altered by CKI in MCF-7 cells. Whether lncRNAs are primary targets or secondary links to regulated pathway, still requires further study. Compared to traditional single-gene analyses used to understand the molecular mechanisms of TCM, transcriptome screening has significant advantages for identifying potential target genes.

Carcinogenesis is a complex cellular process involving multiple genetic alterations that perturb different biological processes or pathways [33]. By screening transcriptome-wide gene expression changes *in vitro*, one can do more than the mere identification of molecular markers for cancer diagnosis or therapy, one can also provide useful evidence to better characterise the underlying mechanisms of drug effects on cancer at pathway or network levels [34, 35]. Using whole transcriptome analysis, we identified multiple potential molecular pathways altered by CKI in MCF-7 cells. Cell growth related pathways, such as cell cycle, cell division, DNA replication and so on, were significantly altered in MCF-7 cells treated with CKI. By integrating the expression data and topologic information of genes involved in these pathways, it appears that cell cycle arrest might be one of the primary anti-tumour mechanisms of CKI in MCF-7 cells. As expected, 5-FU also significantly altered these cell growth related pathways, as reported in previous studies [36, 37]. However, when we consider the expression change of individual genes in these pathways, we found that many different genes were significantly altered by CKI or 5-FU, but the overall perturbation status of these pathways was consistent. We also noticed that CKI and 5-FU had opposite effects on some pathways, such as the p53 signalling pathway. Although the expression of the key *p53* gene in the p53 signalling pathway was significantly down-regulated in cells treated with CKI, we still observed changes in the expression of down-stream genes in the apoptosis pathway, such as *Bcl-2*. *Bcl-2* is an important anti-apoptotic gene [38], which was significantly down-regulated in CKI treated cells, indicating that MCF-7 cells still underwent apoptosis when treated with CKI. The down-regulation of *Bcl-2* had been reported in other types of cancer cells treated with Matrine or Oxymatrine, two of the major components of CKI [16, 39–41]. Taking into account the results from the apoptosis assay, we propose that CKI may induce MCF-7 cell apoptosis via p53 independent pathways. As CKI is normally clinically used in combination with other cancer chemotherapies, our results also provide primary molecular evidence for this potential simultaneous effect in clinical usage [11].

Advances in omics technologies have allowed the development of new cancer therapies [42]. Improved gene therapies, such as targeted cancer therapies or precision medicine, are attracting more and more attention as a result of improved abilities to characterise cancer mechanisms in individual patients [42, 43]. On the other hand, therapies involving whole body system modulation, such as immunotherapy [44] and multiply-targeted therapies [45], are also proposed as potentially effective or complementary weapons for cancer treatment [46]. The nature of many TCMs or ancient medicines that include multiple bioactive ingredients, suggests that they can be a rich resource for identifying or developing multi-targeted cancer drugs [47]. However, most TCMs or ancient medicines are experience-based medicines developed from a long history of clinical use and little is known of their molecular modes of action. Classical pharmacology has used a reductionist strategy of purification and testing of single components from TCM, but this limits our understanding of the potential interaction or cumulative effects of multiple components on a functional system. The application of systems biology techniques, such as whole transcriptome analyses, is a good starting point to understand the functional system effects of TCMs, as a basis for further improvement and optimisation of existing TCMs in the context of evidence based medicine.

Studies have confirmed that many lncRNAs play important regulatory roles in cancer [32]. Our *de novo* identification of lncRNAs showed that some of these may contribute to regulatory networks, and even be specifically or differentially expressed in MCF-7 cancer cells treated by CKI. By integrating lncRNAs with protein-coding RNAs to reconstruct co-expression networks, we showed that this can be used as another powerful tool to understand global transcription changes potentially sensitive to TCM. We were able to confirm that “Cell cycle” and other cell growth related pathways might be the primary target pathways of CKI in MCF-7 cell line as shown in DE analysis, but were able to do this in a more general sense, because we included all expression detectable transcripts during the reconstruction of co-expression networks. In addition, we also showed co-expression networks may be useful in identifying potential co-expressed “hub” transcripts, including both protein-coding RNAs and lncRNAs, for further functional experiments [48]. In conclusion, we applied and integrated multiple transcriptome analysis tools to describe and analyse the complexity of molecular mechanisms altered by CKI in MCF-7 breast cancer cells, and we hope that this can be useful to harness the “magic power” of TCM.

## MATERIALS AND METHODS

### Cell culture and drugs

MCF-7 cells were purchased from ATCC (HTB-22™, VA, USA) and were cultured in DMEM medium (Thermo Fisher Scientific, MA, USA) supplemented with 10% fetal bovine serum (Thermo Fisher Scientific) and 0.01 mg/mL human recombinant insulin (Thermo Fisher Scientific) at 37°C with 5% CO_2_. CKI (total alkaloids concentration of 20.8 mg/mL) was obtained from ZhenDong pharmaceutical Co.Ltd (Shanxi, China), and 5-FU was ordered from Sigma-Aldrich (MO, USA). For all *in vitro* experiments performed in this study, CKI was used at dilution of final concentration of either 1 mg/mL or 2 mg/mL of total alkaloids, and 5-FU was used at a final concentration of 10 ug/mL.

For cell culture in 6-well trays used for cell apoptosis assay, cell cycle assay, p53 protein staining assay and RNA extraction, each well was seeded with 5×10^5^ cells in 2 mL of medium and cultured overnight. On the following day, 1 mL of either medium, CKI or 5-FU was added to the cells. After 24 and 48 hours of treatment, cells were harvested and used in the above assays.

### Cell viability assay

The wells of 96-well trays were seeded with 1×10^4^ cells in 50 μL of medium and cultured overnight. On the following day, 50 μL of either medium, CKI or 5-FU were added to the cells. Viability of the cells was measured at 0, 24 and 48 hours after the treatment by adding XTT:PMS (50:1; Sigma-Aldrich). After 4-hour incubation at 37°C optical density (OD) of each well was read at 490 nm. The background OD was also measured and the average was subtracted from the OD readings of appropriate wells.

### Apoptosis assay by annexin V/PI staining

Cells were cultured in 6-well trays and treated with drugs as described above. After 24 and 48 hours of treatment, cells were harvested and the rate of apoptosis was measured using Annexin V-FITC detecton kit (Biotool, TX, USA) according to the manufacturer's instructions. The stained cells were sorted and data acquired on an LSRII (BD Biosciences, NJ, USA) and the data were analysed using FlowJo software (TreeStar Inc., OR, USA).

### Caspase 3/7 colorimetric assay

Caspase 3/7 activity in cells was measured with a Caspase-3/7 Colorimetric Assay Kit (BioVision, CA, USA). Cells were cultured in 6-well trays and treated with drugs as described above. After 24 and 48 hours of treatment, cells were harvested and proteins from cells were extracted according to the manufacturer's instructions and concentrations were determined with a Nanodrop 2000 (Thermo Scientific). Caspase-3/7 activity was then measured according to the manufacturer's instructions.

### Cell cycle assay

Cells were cultured in 6-well trays and treated with drugs as described above. After 24 and 48 hours of treatment, cells were harvested and subjected to cell cycle analysis by PI staining as described previously [49] and the stained cells were sorted and the data acquired on LSRII and the data were analysed using FlowJo software.

### Intranuclear/intracellular staining for p53

Cells were cultured in 6-well trays and treated with drugs as described above. After 24 and 48 hours of treatment, cells were fixed and permeabilised using Nuclear Factor Fixation and Permeabilization Buffer Set (Biolegend, CA, USA) according to the manufacturer's instructions. 2×10^5^ cells were labelled either with anti-p53-PE or mouse IgG2b-PE (1 μg/mL; Biolegend) and the cells were sorted and the data were acquired on an LSRII, and the data were analysed using FlowJo software.

### RNA extraction and sequencing

Cells were cultured in 6-well plates with a seeding density of 5×10^5^ cells/well and treated with CKI or 5-FU for 24 and 48 hours as above. Total RNA was isolated with the mirVana PARIS Kit (Thermo Fisher Scientific) according to the manufacturer's protocol. RNA samples were sent to the Cancer Genome Facility of the Australian Cancer Research Foundation (SA, Australia) for sequencing. The quality of the total RNA was verified on a Bioanalyzer ensuring all samples had RINs >7.0. Starting with 1 ug of total RNA, the polyA fraction was enriched using a NEBNext(r) Poly(A) mRNA Magnetic Isolation Module. Stranded mRNA libraries for Illumina sequencing were prepared using the NEBNext(r) Ultra Directional RNA kits from New England Biolabs, Inc. according to the manufacturer's protocol (Version 2.0 July 2013). Actinomycin D was added during cDNA synthesis to ensure high levels of strand specificity. All libraries were run on a Bionanalyzer to confirm library size and yield. Barcoded libraries were normalized and pooled based on concentrations determined by qPCR with Library Quantification kits from KAPA Biosystems. Libraries were sequenced using an Illumina HiSeq 2500 across 5 lanes with stranded paired-end 100 base pair reads. Raw and processed data were deposited at the Gene Expression Omnibus (GEO) data repository (GSE78512).

### Data processing and functional annotation

Low quality and adaptor sequences in raw reads were trimmed using Trim_galore (v0.3.7, Babraham Bioinformatics) with the following parameters: --stringency 6 --paired. Then cleaned reads were aligned to the reference genome (hg19, UCSC) using STAR_2.4.0j with the following parameters: --outSAMstrandField intronMotif --outSAMattributes All --outFilterMismatchNmax 10 --seedSearchStartLmax 30 [50]. Differential expression analysis was performed with edgeR and DE genes were selected with a False Discovery Rate (FDR) < 0.05 [51].

GO and KEGG over-representation analyses were performed using ClueGO with the following settings: biological process at 3rd level (for GO); right-sided hypergeometric test for enrichment analysis; p values were corrected for multiple testing according to the Benjamini-Hochberg method. Over-represented terms/pathways were visualised with Cytoscape v3.2.1 [52, 53]. Signalling Pathway Impact Analysis (SPIA) was performed with SPIA package in R [19]. Gene expression status mapping in KEGG pathways was visualised with the R Pathview package [54].

### Transcriptome validation with qPCR

Cells were cultured in 6-well trays and treated with drugs as described above. Untreated cells as well as cells treated for 24 and 48 hours were harvested and the cell pellets were snap-frozen in liquid nitrogen. Total RNA was extracted using PureLink RNA Mini Kit (Thermo Fisher Scientific) and treated with TURBO DNA-Free (Thermo Fisher Scientific) according to the manufacturer's instructions. cDNA synthesis was performed using High Capacity cDNA Reverse Transcription Kit (Thermo Fisher Scientific) according to the manufacturer's instructions.

qPCR reactions were set up with PowerUp SYBR Green Master Mix where forward and reverse primers were added at a final concentration of 400 nM each. Reactions were run on the StepOne Plus Real-Time PCR system and the data were analysed using its software v2.3 (Thermo Fisher Scientific). Relative levels of target mRNAs were calculated as 1/2^ΔCT^, where ΔCT=CT of target − CT of *RSP13*. The sequences of all primers used in this study are provided in Supplementary Table 6.

### LncRNA identification

The flowchart for lncRNA identification is shown in Supplementary Figure 6. In summary, short reads were mapped against the genome and assembled into longer transcripts, and then transcripts shorter than 200 nucleotides (nt) were removed. Genomic coordinates of long transcripts were checked against refGenes from UCSC and classified into “refGene transcripts”, “intergenic transcripts”, “intronic transcripts” and “antisense transcripts”. The latter three classes of transcripts were selected to filter unannotated protein-coding potential transcripts by following two steps: 1) Sequence similarity search against the Swiss-Prot protein database; 2) Predict Open Reading Frame(s) (ORF). In order to get a more reliable lncRNA dataset, we selected transcripts with expression higher than 1 count per million (CPM, normalised using the TMM method in edgeR) in at least 2 of 27 individual samples.

### Reconstruction of co-expression networks

RefGenes were pre-filtered by expression (> 1 CPM in at least 2 of 27 individuals). Expression matrices for all pre-filtered refGenes and lncRNAs were merged to reconstruct co-expression networks with WGCNA [55]. “16” was selected as the soft thresholding power according to the protocol of WGCNA. Co-expression modules were visualized with Cytoscape v3.2.1. Between-ness centrality was used to select “hub” nodes. GO and KEGG over-representation analyses were performed with DAVID (Database For Annotation, Visualization and Integrated Discovery) [56].

## SUPPLEMENTARY MATERIALS FIGURES AND TABLES














