# Supplementary material for: Identification of candidate anti-cancer molecular mechanisms of Compound Kushen Injection using functional genomics
Source: Oncotarget. 2016 Sep 1;7(40):66003–19. doi: 10.18632/oncotarget.11788 (PMC5323210; doi:10.18632/oncotarget.11788)
Supplement: Supplementary file 1 [file oncotarget-07-66003-s001.pdf]

Identification of candidate anti-cancer molecular mechanisms of compound kushen injection using functional genomics

SUPPLEMENTARY FIGURES AND TABLES

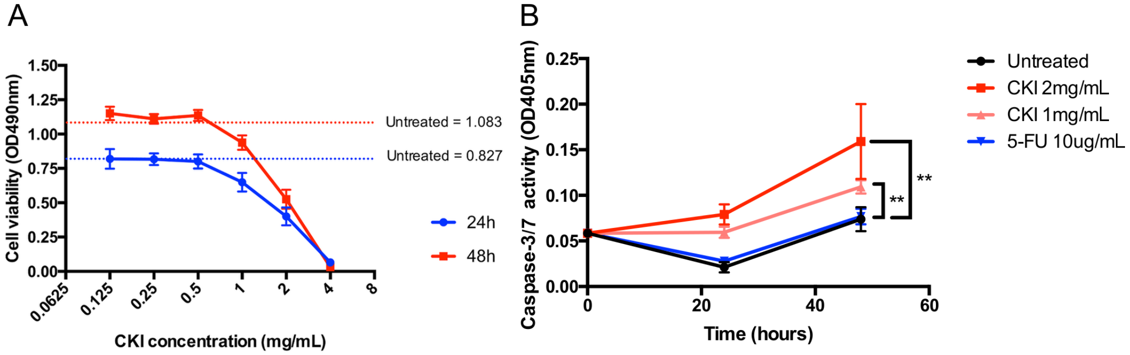

Supplementary Figure 1: CKI inhibited MCF-7 cell growth in a dose-dependent fashion and increased caspase-3/7 activity. A. The level of viability of cells after different treatments were measured using XTT:PMS. Data are represented as mean ±SEM (n=9). X-axis is log2 scaled. **B.** The level of caspase-3/7 activity in cells was measured with Caspase-3/7 Colorimetric Assay Kit. Data are represented as mean ±SEM (n=3). Statistical analyses were performed using two-way ANOVA (**p<0.01, ***p<0.001).

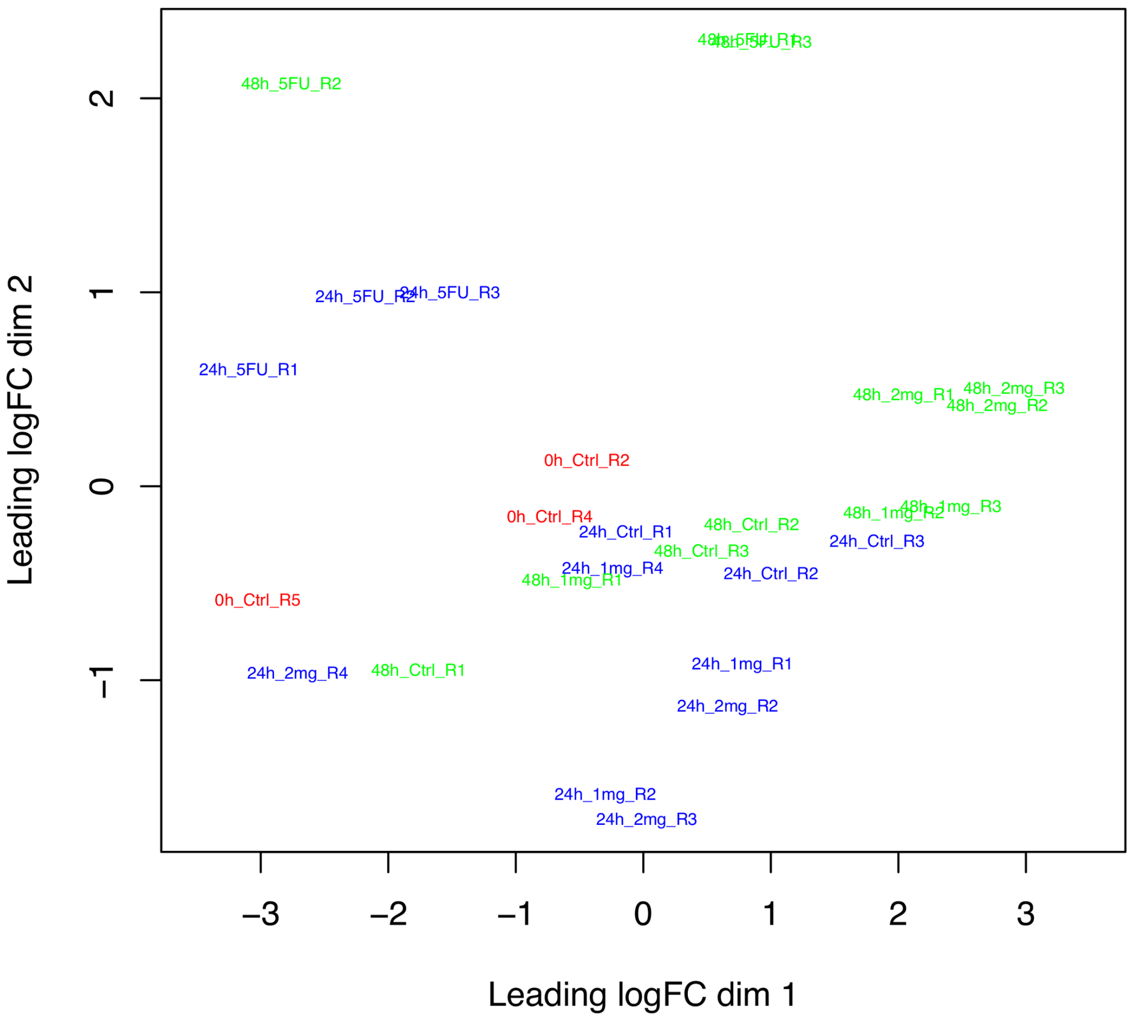

Supplementary Figure 2: Multiple dimensional scaling (MDS) plot for samples based on expression profiles of all genes.

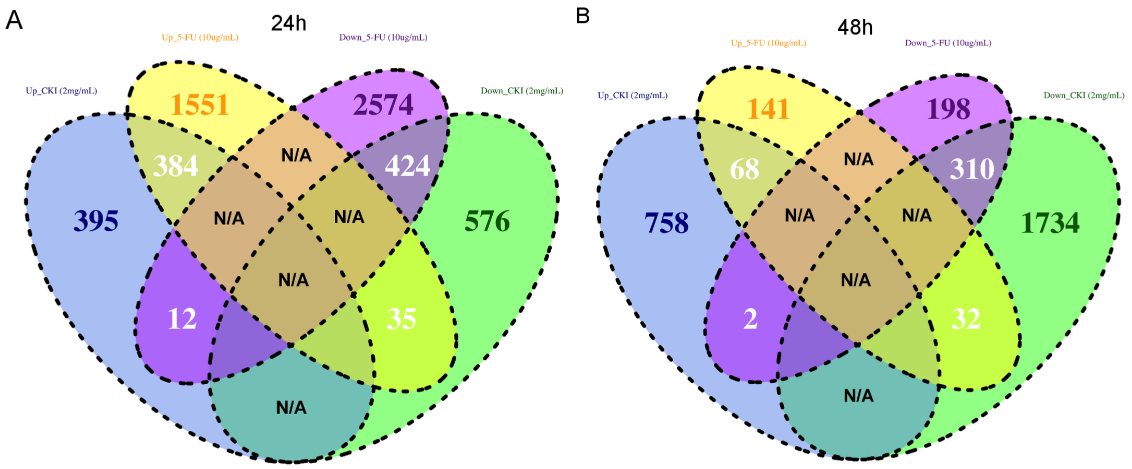

Supplementary Figure 3: Venn diagrams showing the overlap of DE genes in cells treated with CKI (2 mg/mL) or 5-FU for A. 24 hours or **B.** 48 hours.

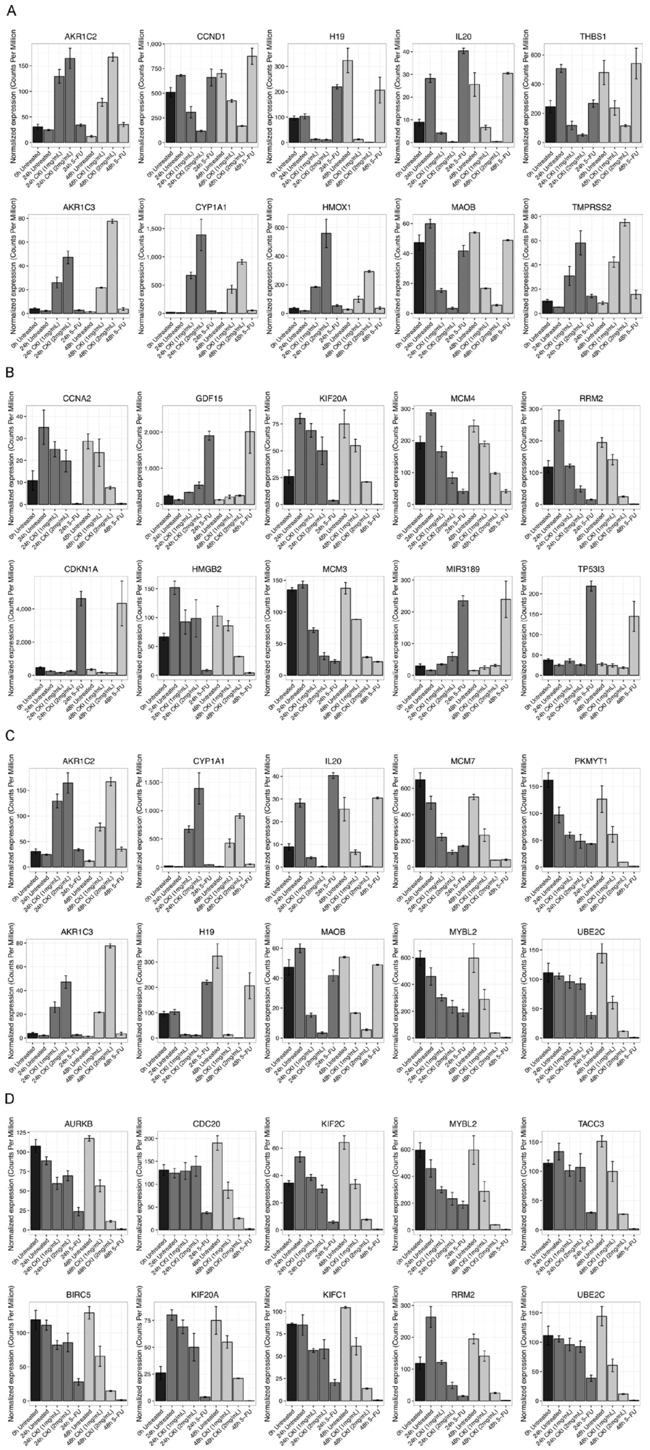

Supplementary Figure 4: Normalised expression values of the top10 significantly differentially expressed genes in cells treated with CKI (2 mg/mL) or 5-FU for 24 hours A. and **B.** or 48 hours **C.** and **D.**

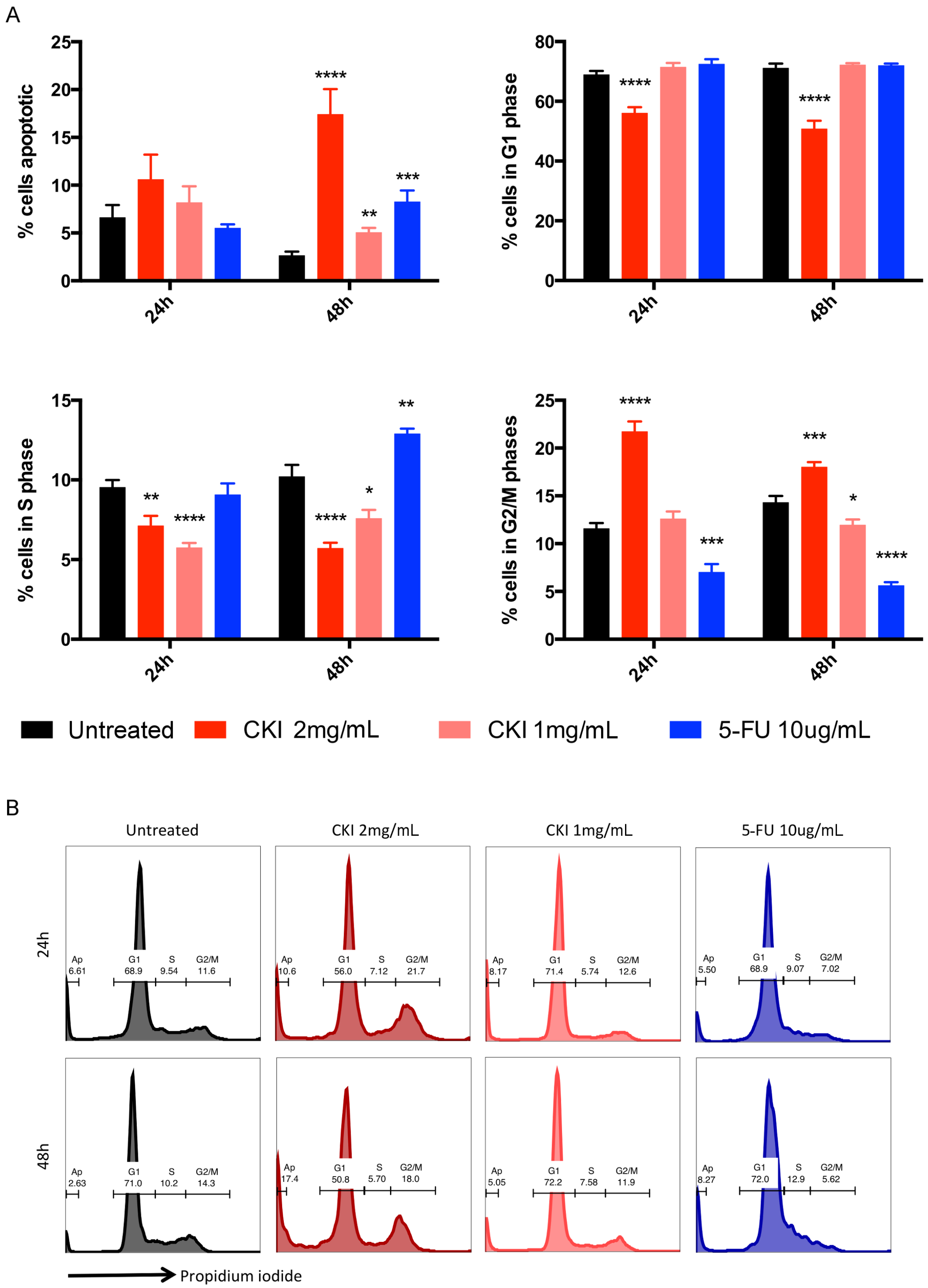

Supplementary Figure 5: Effect of CKI on cell cycle and apoptosis in MCF-7 cells. Proportions of cells in either apoptosis, G1, S or G2/M phase were determined by measuring the DNA contents of permeabilised cells stained with Propidium Iodide. **A.** Percentages of cells in each cell cycle phase. **B.** Representative histograms of PI staining with gating designation. Data are represented as mean ±SEM (n=9). Statistical analyses were performed using t-test comparing with "Untreated" (*p<0.05, **p<0.01, ***p<0.001, ****p<0.0001).

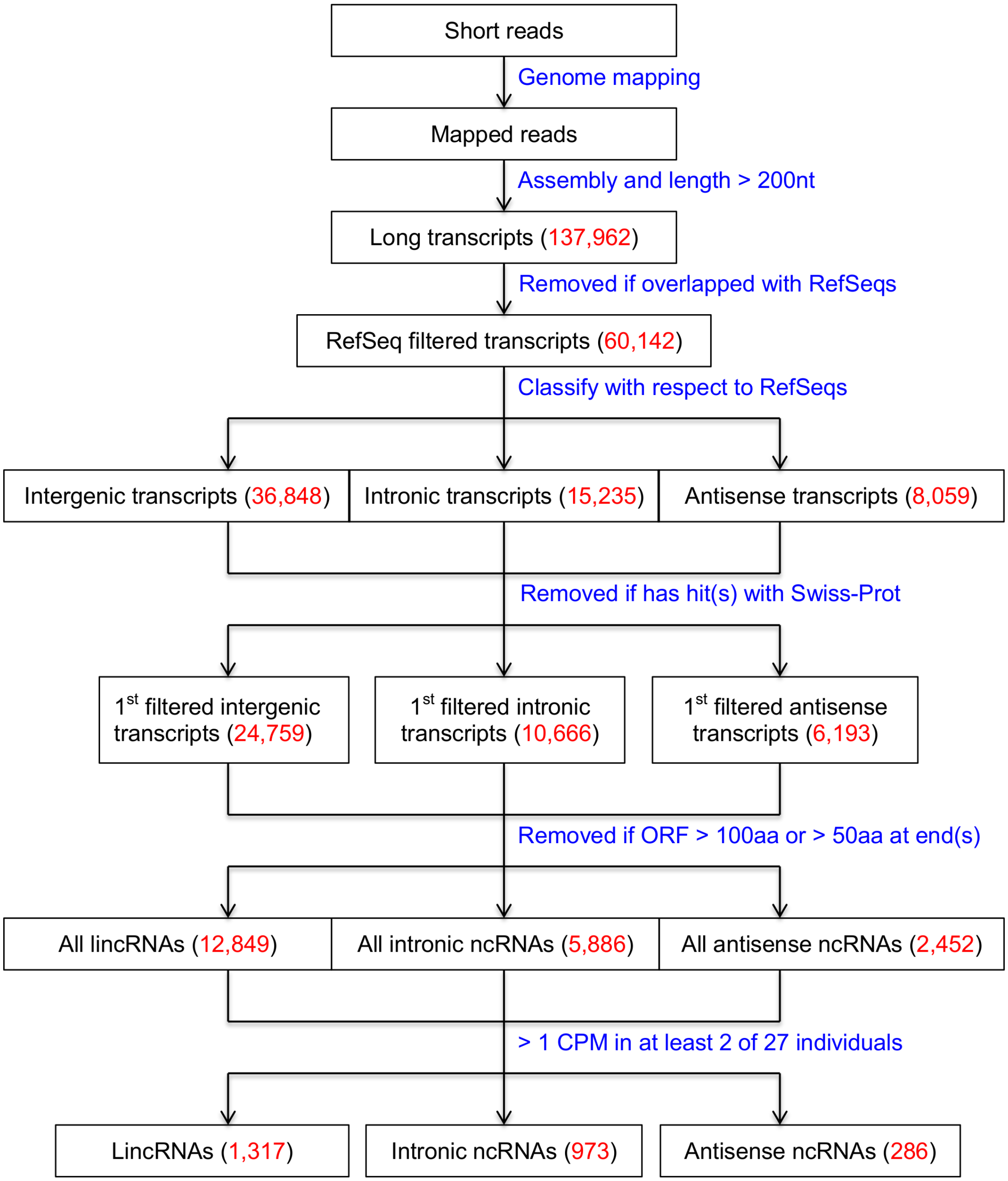

Supplementary Figure 6: Flowchart of *de novo* identification of lncRNAs from RNA-seq dataset. Digits in red colour represent the numbers of transcripts after corresponding data processing.

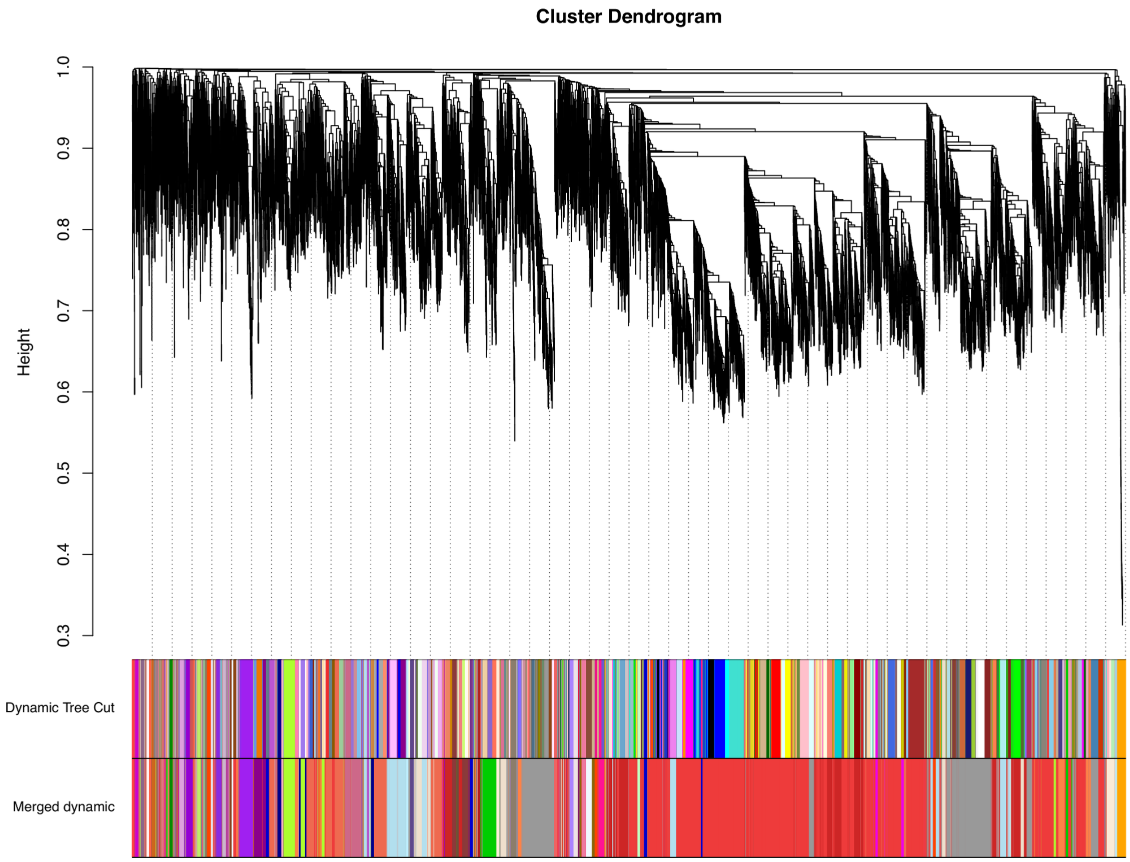

Supplementary Figure 7: Clustering dendrogram of genes, with dissimilarity based on topological overlap, together with co-expression module colors. Merged dynamic modules (lower color band) mean highly co-expressed modules are merged based on eigengene correlations in original clustered modules (upper color band).

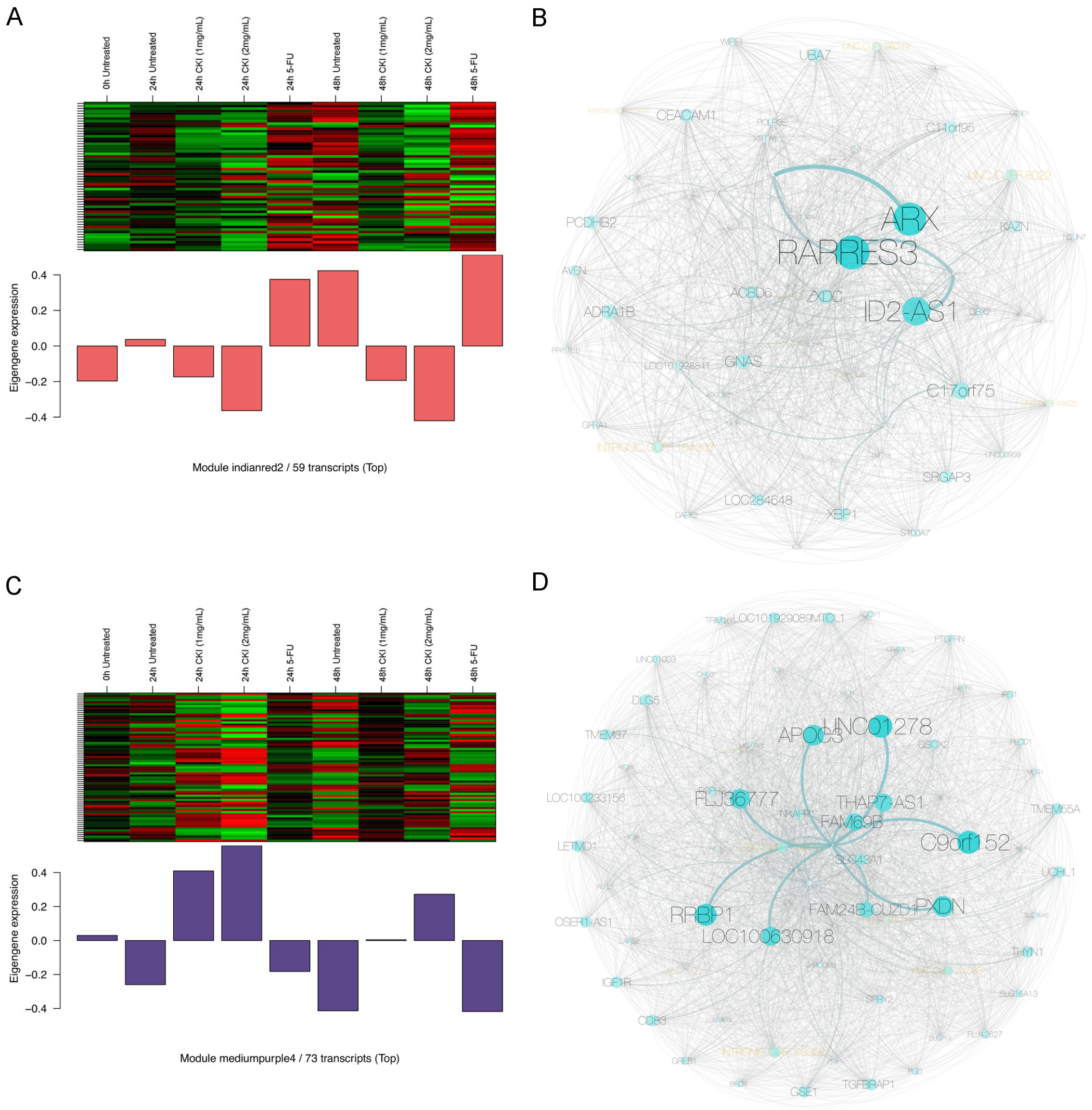

Supplementary Figure 8: Expression patterns of transcripts in CKI-specific modules "indianred2" and "mediumpurple2" are shown in the top panels A. and **C.** and the barplot in the bottom panels (A and C) shows the eigengene values for different samples. Green represents "under-expressed" and red represents "over-expressed" in the heatmap. The "eigengene value" is defined as the first principal component of this module, so it can be considered as representative of the gene expression profiles in this module. Visualization of CKI-specific modules "indianred2" and "mediumpurple2" **B.** and **D.** The black labels represent refGenes and gold labels represent lncRNAs. The size of the node/label and edge weight is proportional to betweenness centrality.

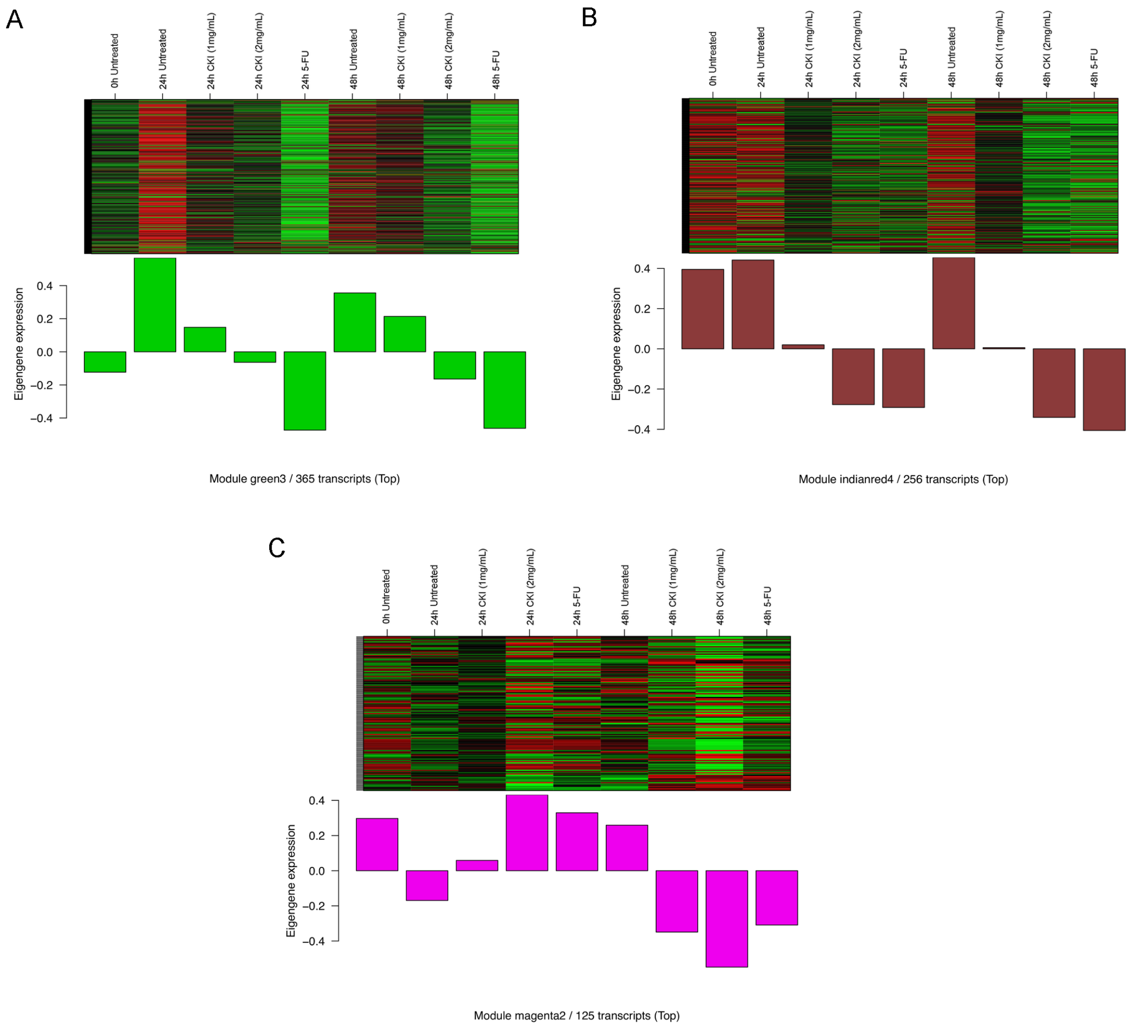

Supplementary Figure 9, Representative CKI-5FU co-expression modules. Expression patterns of transcripts in CKI-5FU modules **A.** "green3", **B.** "indianred4" and **C.** "magenta2" are shown in the top panels, and the barplot in the bottom panels show the eigengene values in different samples. Green represents "under-expressed" and red represents "over-expressed" in the heatmap. The "eigengene value" is defined as the first principal component of this module, so it can be considered as representative of the gene expression profiles in this module.

Supplementary Table 1: Summary of RNA-seq datasets used in this study

See Supplementary File 1

Supplementary Table 2: Summary of significantly differentially expressed genes for different comparisons

See Supplementary File 2

Supplementary Table 3: Significantly perturbed KEGG pathways based on SPIA analysis

See Supplementary File 3

Supplementary Table S4: Numbers of transcripts in 53 co-expression modules

See Supplementary File 4

Supplementary Table 5: Significantly over-represented GO and KEGG terms in protein-coding genes from three CKI-5FU co-expression modules (count > 4 and P-value < 0.05)

See Supplementary File 5

Supplementary Table 6: Primers used for qPCR

See Supplementary File 6
